# 12-month prevalence of asthma among adults in Germany

**DOI:** 10.17886/RKI-GBE-2017-064

**Published:** 2017-10-09

**Authors:** Henriette Steppuhn, Ronny Kuhnert, Christa Scheidt-Nave

**Affiliations:** Robert Koch Institute, Department of Epidemiology and Health Monitoring, Berlin

**Keywords:** ASTHMA, PREVALENCE, ADULTS, HEALTH MONITORING, GERMANY

## Abstract

Asthma is a chronic inflammatory disease of the airways affecting people of all ages. The disease is characterised by a variable narrowing of the bronchia, which may be accompanied by symptoms such as wheezing or shortness of breath. In GEDA 2014/2015-EHIS, 6.2% of respondents aged 18 years or older with complete information on the respective indicator (n=22,671) reported having had asthma during the past 12 months. The 12-month prevalence among women (7.1%) is higher than among men (5.4%). Overall, women and men with a low level of education more often reported having had asthma than those with a higher level of education. In analyses stratified by age and gender, differences in asthma prevalence with regard to educational level are evident among women under 30 years of age. In a comparison of federal states, the prevalence of asthma ranges from 3.0% to 9.7% among women and from 2.9% to 7.0% among men.

## Introduction

According to the World Health Organization (WHO), asthma is one of the most common chronic diseases affecting around 235 million people globally [1]. Asthma can occur among people of all ages [2-5]. The disease is characterised by chronic inflammation and increased sensitivity (hyperresponsiveness) of the airways to diverse inhaled stimuli [5, 6]. Asthma patients moreover suffer from variable narrowing of the bronchia (airway obstruction) accompanied by symptoms such as wheezing, shortness of breath, chest tightness or coughing which vary over time and in intensity [5, 6]. Some patients with mild asthma may experience periods when they are completely free of symptoms [5, 7]. In particular, in case of an asthma onset in childhood, remission of symptoms during puberty is frequently observed [4, 8].

Asthma is a heterogeneous disease which results from different underlying disease mechanisms [5, 9]. Allergic asthma and also other forms of non-allergic asthma are marked by a characteristic type of inflammation [5, 9]. This (TH2) type of inflammation is indicated by elevated concentrations of specific immune cells (eosinophils) in the airway mucosa and in the blood (eosinophilic asthma) [5, 6, 9]. Further, mainly adult-onset, forms of asthma exist, where such an overproduction of eosinophils cannot be observed (non-eosinophilic asthma) [5, 6, 9]. It is important to distinguish between eosinophilic and non-eosinophilic asthma, when treating patients with severe asthma [5, 6, 9].

A family history of asthma or of certain allergies (such as hay fever) is an important predisposing factor [5, 10-12]. Asthma thereby results from complex interactions between genetic and environmental factors [2, 5, 12]. Early childhood yet also prenatal influences are highly relevant in the induction of the disease [2, 5, 12]. Infections, exposure to microbes, pollutants and allergens, but also diet or psychosocial factors may contribute to asthma development [2, 5, 12]. Furthermore, occupational exposures have been identified (e.g., allergens such as flour and cow hair or chemical irritants such as disinfectants and agents used by hairstylists) which can induce or aggravate asthma in adulthood [2, 13]. Providing appropriate medical care to asthma patients is highly important to prevent episodes in which the disease worsens acutely and which can require emergency care or lead to premature mortality [5]. In the majority of patients, however, adequate treatment can minimise the symptoms of asthma and associated limitations in usual activities [5].

**GEDA 2014/2015-EHIS**

**Data holder:** Robert Koch Institute

**Aims:** To provide reliable information about the population’s health status, health-related behaviour and health care in Germany, with the possibility of a European comparison

**Method:** Questionnaires completed on paper or online

**Population:** People aged 18 years and above with permanent residency in Germany

**Sampling:** Registry office sample; randomly selected individuals from 301 communities in Germany were invited to participate

**Participants:** 24,016 people (13,144 women; 10,872 men)

**Response rate:** 26.9%

**Study period:** November 2014 - July 2015

**Data protection:** This study was undertaken in strict accordance with the data protection regulations set out in the German Federal Data Protection Act and was approved by the German Federal Commissioner for Data Protection and Freedom of Information. Participation in the study was voluntary. The participants were fully informed about the study’s aims and content, and about data protection. All participants provided written informed consent.

More information in German is available at

www.geda-studie.de

## Indicator

In the GEDA 2014/2015-EHIS survey, the prevalence of asthma during the past 12 months was assessed by using a self-administered paper-based or online questionnaire. Respondents were asked, ‘During the past 12 months, have you had any of the following diseases or conditions?’ This question was followed by a list of conditions that also contained ‘asthma (allergic asthma included)’. 24,016 adults aged 18 years or older (13,144 women and 10,872 men) participated in GEDA 2014/2015-EHIS. 1,345 respondents (696 women and 649 men) with missing information on the indicator on self-assessed asthma were excluded from the current analysis. The calculations were carried out using a weighting factor that corrects for deviations within the sample from the German population structure (as of 31 December 2014) with regard to gender, age, district type and education. The district type reflects the degree of urbanisation and corresponds to the regional distribution in Germany. The International Standard Classification of Education (ISCED) was used to classify the responses provided on educational level [14]. Lange et al. [15] set out the details of the methodology applied in the GEDA 2014/2015-EHIS study including the method used to calculate the weighting factor and an assessment of the response rate. Background information on the GEDA 2014/15-EHIS is also provided in the article German Health Update: New data for Germany and Europe in Issue 1/2017 of the Journal of Health Monitoring.

## Results and discussion

**Figure 1:**
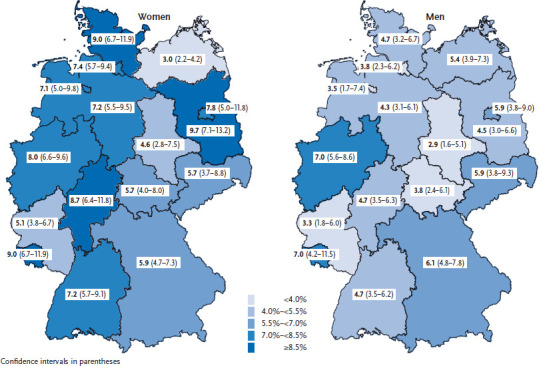
12-month prevalence of asthma by gender and federal state (n=12,448 women; n=10,223 men) Source: GEDA 2014/2015-EHIS

6.2% of adults aged 18 years or older reported having had asthma during the past 12 months. Overall, women (7.1%) show a higher 12-month prevalence of asthma than men (5.4%) (Table 1). Asthma prevalence is highest in the age group 45 to 64 years among men while it remains on a similar level across all age groups among women. Overall, women and men with a low level of education more frequently reported to have had asthma than those with a medium or high level of education (7.7% vs. 5.9% and 5.7%). In analyses stratified by age and gender, these differences with regard to educational level are evident among women aged 18 to 29 years (Table 1). Prevalence estimates vary considerably between federal states ranging from 3.0% in Mecklenburg Western-Pomerania to 9.7% in Brandenburg among women and from 2.9% in Saxony-Anhalt to 7.0% in North Rhine Westphalia and Saarland among men (Figure 1).

The current results on the 12-month prevalence of asthma in GEDA 2014/2015-EHIS are consistent with results of the German national telephone health interview survey German Health Update (GEDA 2012) conducted by the Robert Koch Institute in 2012 [16]. This applies both to the overall prevalence of asthma among adults (6.2% vs. 6.3% in 2012) and to estimates by gender (women: 7.1% vs. 7.5%; men: 5.4% vs. 5.0%) [16]. Comparable differences between genders (an overall 1.3 to 1.5-fold higher prevalence of asthma among women) were observed in epidemiological surveys in Europe, Australia and the USA [17]. Results from longitudinal studies moreover point to an increased asthma risk for women relative to men, whereby this difference is already evident in adolescence (see also the Focus article on respiratory diseases in this issue) [17, 18]. Regarding socio-economic differences, the surveys available revealed heterogeneous results. Overall, in the majority of studies, however, a higher prevalence of asthma is related to lower social status [19]. A comparison of GEDA 2014/2015-EHIS results by gender or socio-economic criteria needs to consider further aspects. Differences in the way patients perceive symptoms, seek medical care or adhere to treatment recommendations - and resulting differences in the degree of treatment and asthma control - may all affect levels of self-reported asthma prevalence [20, 21].

Direct comparisons of prevalence estimates obtained from GEDA 2014/2015-EHIS with those obtained from previous German national health surveys need to consider some change in methods and hence preclude analyses of trends in asthma prevalence over time [16, 22-24]. In accordance with the regulation on the harmonisation of European health reporting, GEDA 2014/2015-EHIS used self-reported information on asthma prevalence based on the self-assessment of respondents. As in other large epidemiological surveys [25-34], all national health surveys previously conducted in Germany, starting with the German Health Interview and Examination Survey 1997-1999 (GNHIES98) [16, 22-24], however, collected data on self-reported physician-diagnosed asthma. In comparison with the currently applied indicator on self-assessed asthma, prior results on the prevalence of physician-diagnosed asthma may be less prone to misclassification [32]. In addition, previous national health surveys also assessed the lifetime prevalence of physician-diagnosed asthma which was consistently higher than the recorded 12-month prevalence [16, 22-24]. These differences between lifetime and 12-month prevalence indicate the varying degrees of disease activity over time in individuals with asthma [4, 7, 8]. In some patients, symptoms of asthma may resolve, at least for longer periods of time [4, 7, 8].

In Germany, an increase in the lifetime prevalence of physician-diagnosed asthma was recorded for both genders among adults (1997-1999 and 2008-2011) as well as among children and adolescents (2003-2006 and 2009-2012) [22-24]. At the same time, an increment in the 12-month prevalence was likewise observed; among adults this was attributable mainly to an increase in the prevalence among women [23, 35]. Interview survey data had already indicated a rising prevalence of asthma in the 1990s in Germany especially among children [26, 29, 36-40]. Moreover, an increase in the prevalence of asthma was also observed in other parts of the world during the second half of the last century [29]. Recent data indicates that this trend has come to a halt at least in certain regions such as Australia where prevalence is already high [25, 29, 41]. Among adults, however, a further increase in prevalence over the course of the last decade has nonetheless been recorded in several countries in Europe and in the USA [27, 28, 31, 33, 42].

High regional variation in asthma prevalence has been observed not only in GEDA 2014/2015-EHIS based on analyses stratified by federal state. Using ambulatory health care data, a recent study reported considerable variation in asthma prevalence at the county level ranging from 2.5% to 7.7% for the total population [43]. Earlier European surveys indicated significant regional differences with a high prevalence mainly in the United Kingdom and low prevalence in Eastern European regions [2, 3, 44, 45]. In line with Europe-wide data for adults [2, 46], current asthma prevalence in Germany as recorded in GEDA 2014/2015-EHIS is mid-range compared to other countries of the EU. Previous studies including a national health survey of adults in Germany indicated a lower prevalence of asthma in East than in West Germany [47-51]. The German Health Interview and Examination Survey for Children and Adolescents (KiGGS) 2003-2006, however, which was conducted among children and adolescents most of whom were born after German reunification, revealed no significant differences between East (including Berlin) and West Germany [24]. Future investigations on the regional differences in asthma prevalence among adults in Germany should thus include in-depth analyses that do not only stratify by age and birth year, respectively, but also consider the place where respondents were born and raised.

Previously observed temporal and regional differences in asthma prevalence were related to the development of time trends and the geographical distribution of several lifestyle and environmental factors [12, 29, 50]. In addition, the influence of temporal changes and regional differences in degrees of awareness and provision of care, respectively, was also discussed [27, 29, 43, 50]. The recently observed changes in asthma prevalence over time point to the importance of continuous health monitoring based on periodically repeated national health surveys using comparable instruments of data collection. Besides extending the available database from national health interview and examination surveys permitting analyses of trends in the prevalence of physician-diagnosed asthma, it will be highly important to build a reliable database for the analysis of trends in self-assessed asthma prevalence using the current indicator in the context of European health interview surveys. Moreover, clarifying the underlying causes of regional and gender-specific differences in the prevalence of asthma remains a key challenge [12].

## Key statements

- About 6% of adults reported having had asthma during the past 12 months.
- Women (7.1%) show a higher 12-month prevalence of asthma than men (5.4%).
- In a comparison of federal states, the prevalence of asthma ranges from 3.0% to 9.7% among women and from 2.9% to 7.0% among men.

## Figures and Tables

**Table 1:** 12-month prevalence of asthma by gender, age and educational level (n=12,448 women; n=10,223 men) Source: GEDA 2014/2015-EHIS

Women	%	(95% CI)	Men	%	(95% CI)
**Women total**	**7.1**	**(6.5-7.7)**	**Men total**	**5.4**	**(4.8-5.9)**
**18-29 Years**	7.8	(6.4-9.6)	**18-29 Years**	3.6	(2.7-4.7)
Low education	13.3	(8.9-19.4)	Low education	4.1	(2.1-7.7)
Medium education	6.5	(5.1-8.2)	Medium education	2.9	(2.0-4.1)
High education	5.2	(3.3-8.2)	High education	5.8	(3.5-9.5)
**30-44 Years**	6.4	(5.4-7.6)	**30-44 Years**	5.1	(4.1-6.4)
Low education	8.5	(5.4-13.1)	Low education	7.3	(4.1-12.6)
Medium education	6.1	(4.8-7.6)	Medium education	4.4	(3.1-6.1)
High education	6.2	(4.8-8.0)	High education	5.7	(4.0-8.1)
**45-64 Years**	7.1	(6.3-8.0)	**45-64 Years**	6.3	(5.4-7.2)
Low education	7.0	(5.0-9.6)	Low education	8.5	(5.8-12.3)
Medium education	7.1	(6.1-8.4)	Medium education	6.6	(5.3-8.1)
High education	7.2	(5.8-8.8)	High education	5.0	(4.0-6.2)
**≥ 65 Years**	7.1	(6.1-8.4)	**≥ 65 Years**	5.5	(4.5-6.7)
Low education	7.3	(5.6-9.5)	Low education	8.0	(5.3-11.9)
Medium education	6.7	(5.2-8.5)	Medium education	5.3	(3.9-7.0)
High education	6.9	(4.7-10.1)	High education	4.8	(3.4-6.7)
**Total (women and men)**	**6.2**	**(5.8-6.7)**	**Total (women and men)**	**6.2**	**(5.8-6.7)**

CI=Confidence interval

* n=51 additional missing values (26 women, 25 men) when stratifying by educational level
